# Biosensors in Clinical Practice: Focus on Oncohematology

**DOI:** 10.3390/s130506423

**Published:** 2013-05-14

**Authors:** Nicola S. Fracchiolla, Silvia Artuso, Agostino Cortelezzi

**Affiliations:** 1 Department of Hematology-Oncology and BMT Unit, IRCCS Cà Granda Ospedale Maggiore Policlinico, Via Francesco Sforza 35, 20122 Milan, Italy; E-Mails: silvia.artusos@libero.it (S.A.); agostino.cortelezzi@unimi.it (A.C.); 2 Faculty of Medicine, University of Milan, Via Francesco Sforza 35, 20122 Milan, Italy

**Keywords:** biosensors, leukemia, oncohematology, clinical applications

## Abstract

Biosensors are devices that are capable of detecting specific biological analytes and converting their presence or concentration into some electrical, thermal, optical or other signal that can be easily analysed. The first biosensor was designed by Clark and Lyons in 1962 as a means of measuring glucose. Since then, much progress has been made and the applications of biosensors are today potentially boundless. This review is limited to their clinical applications, particularly in the field of oncohematology. Biosensors have recently been developed in order to improve the diagnosis and treatment of patients affected by hematological malignancies, such as the biosensor for assessing the *in vitro* pre-treatment efficacy of cytarabine in acute myeloid leukemia, and the fluorescence resonance energy transfer-based biosensor for assessing the efficacy of imatinib in chronic myeloid leukemia. The review also considers the challenges and future perspectives of biosensors in clinical practice.

## 1. Introduction

The International Union of Pure and Applied Chemistry defines a biosensor as “a device that uses biochemical reactions mediated by isolated enzymes, organelles or whole cells to detect the effects of chemical compounds by electrical, thermal or optical signals” [1].

Newman *et al*. have proposed defining a biosensor as “a compact analytical device incorporating a biological or biologically-derived sensing element either integrated within or intimately associated with a physicochemical transducer; the usual aim of such a device is to produce either a discrete or continuous digital electronic signal that is proportional to a single analyte or a related group of analytes” [2].

**Figure 1.**
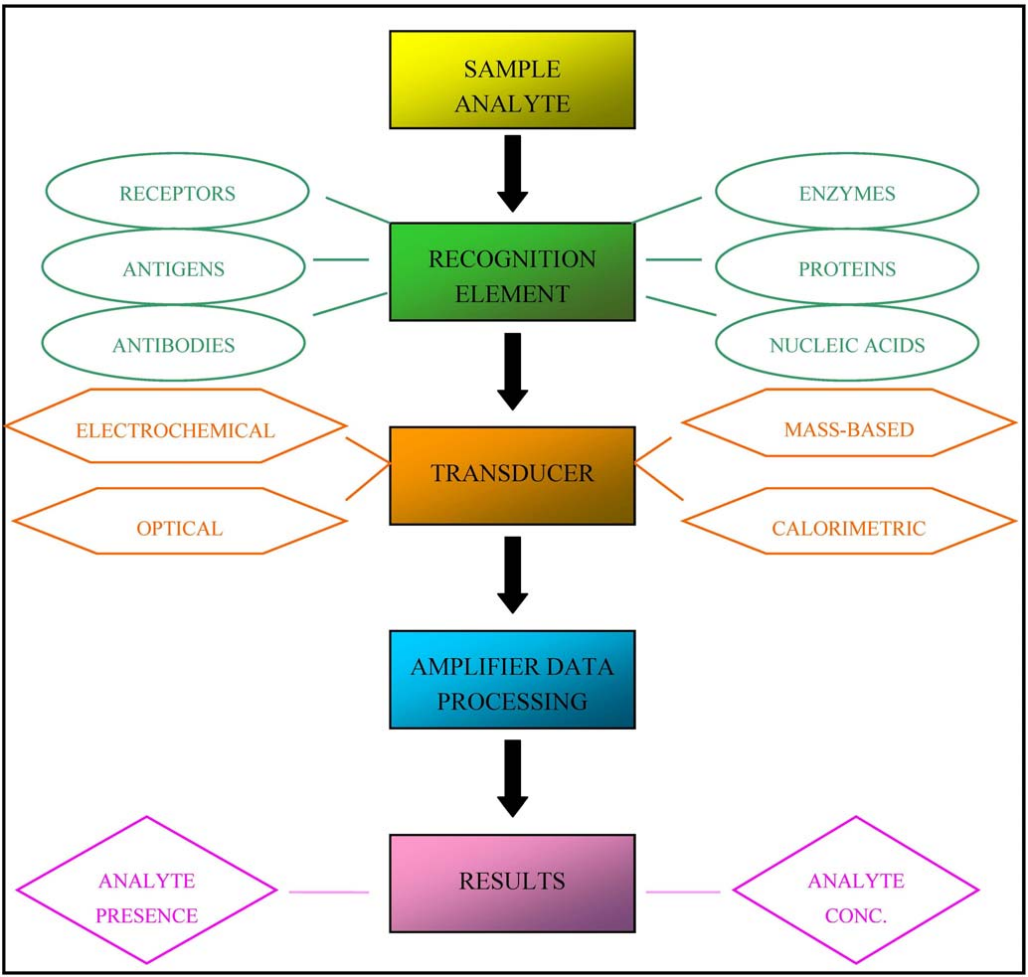
Basic structure and functioning of biosensors.

In practice, biosensors are devices that are capable of detecting specific biological analytes and converting their presence or concentration into signals that can be easily detected and analysed. All biosensors can be divided into three main components, as illustrated in Figure 1: a recognition element (for example antibodies, receptor proteins, nucleic acids, antigens or enzymes) that detects the “signal” from the environment in the form of an analyte; a signal transducer that converts the biological signal into an electronic output; and a signal processor that relays and displays the results [3].

Biosensors can be classified into four classes on the basis of the type of transducer: optical (colorimetric, fluorescent, luminescent, and interferometric) biosensors, mass-based (piezoelectric and acoustic wave) biosensors, calorimetric, and electrochemical (amperometric, potentiometric and conductometric) biosensors [4]. Alternatively, they can be classified on the basis of the nature of recognition [5].

Optical biosensors measure the light observed or emitted as a result of a biological and/or chemical reaction [6], and can be further categorised as being based on luminescence, fluorescence, colorimetry or interferometry [7]. They are very sensitive and, for example, can be used for the pre-treatment screening of effective drug doses [3]. In particular, the selectivity and sensitivity of fluorescence mean that it is often used for biosensing applications. Fluorescence-based biosensors measure the change in frequency of electromagnetic radiation emission (caused by previous absorption of radiation and the generation of an excited state), and the repeated excitation of single molecules produces a bright signal that can be measured even at single-cell level [8].

Mass-based devices include piezoelectric and acoustic wave biosensors [3]. Piezoelectric biosensors have a piezoelectric component (usually a quartz-crystal coated with gold electrodes) that can be made to oscillate at a specific frequency by applying an electrical signal [8], and their surface is coated with a biologically active substance; when they are positioned in a solution containing analytes that bind to the active substance, the mass of the system increases and the resonance frequency of oscillation proportionally decreases [6]. Similarly, mass loading on the surface of acoustic wave biosensors leads to a shift in output frequency that can be used to measure analyte concentration indirectly [9]. They are very simple to use and cheap, but lack specificity, selectivity and sensitivity [6].

Calorimetric (or thermometric) biosensors, which are less common than other types, measure changes in heat due to exothermic reactions between the biomolecule immobilised on temperature sensors and the analyte; changes in enthalpy can be indirectly used to determine analyte concentrations [3]. They do not require frequent recalibration, are insensitive to the optical and electrochemical properties of the sample, and have been used to analyse food and pharmaceutical cosmetics [8].

Electrochemical biosensors measure the electrochemical signal produced during a biochemical interaction between a biologically active substance and a substrate in which electrochemical species are consumed or generated. They can be further differentiated into potentiometric, amperometric and conductometric biosensors depending on the electrochemical property they measure [6]. The first two are the most frequently used: potentiometric biosensors have ion-selective electrodes that detect an electrical response in the molecular recognition element, whereas amperometric transducers measure the current produced by the potential placed between two electrodes [3]. One of the two electrodes (the “working electrode”) is usually a noble metal or a screen-printed layer covered by a bioelement. Conductometric (or impedimetric) biosensors measure the changes in electrical conductance or resistance of a solution determined by the changes in function of the ions or electrons produced during the course of a biochemical reaction between the biocomponent and the analyte [8].

Enzymes, antigens/antibodies, cells and viruses, nucleic acids, or biomimetic materials can be used as biosensor biocomponents for analyte recognition. Enzyme-based biosensors use the catalytic and binding capacities of enzymes for specific detection, and are much more sensitive than other biosensors; the products of reactions catalysed by enzymes can be detected directly or by means of a marker. Antigen/antibody-based biosensors use the highly specific binding between an antigen and its antibody; this binding (which, for example, can be detected by means of fluorescent labelling) must occur under conditions in which non-specific interactions are minimised. Cell- and virus-based biosensors use some features of micro-organisms (such as bacteria metabolism or bioluminescence) to detect specific molecules. Nucleic acid-based biosensors use the specific complementary relationships of DNA base pairs (adenine-thymine and guanine-cytosine) and, for example, can detect small amounts of bacterial DNA by hybridising it with a complementary strand of known DNA. Biosensors based on biomimetic materials are artificial or synthetic devices that mimic the function of natural biosensors: one example is an aptasensor, in which the biocomponent consists of aptamers (synthetic strands of nucleic acids that can be designed to recognise a specific analyte) [5].

The surface immobilisation of biocomponents, which is usually done by means of physical adsorption, covalent binding, matrix entrapment, inter molecular cross-linking or membrane entrapment, is an important step in producing a biosensor because it influences performance [8]. The main technologies used in biosensor functioning are surface plasmon resonance, fluorescence resonance energy transfer, and bioluminescence resonance energy transfer.

### 1.1. Surface Plasmon Resonance

Surface plasmon resonance (SPR), which was first developed by Liedeberg *et al*. in 1983, is widely used in optical biosensing applications [10]. SPR biosensors rely on an optical phenomenon occurring at the interface between a free electron-rich metal (gold, silver, *etc.*) and a dielectric medium, classically either a liquid or air [11]: when an appropriate wave vector of light resonates with the free electrons in a metal, electromagnetic waves (known as surface plasmons) appear on the metal's surface and attenuate the intensity of light. The resonant condition depends on the refractive index in the vicinity of the metal surface and, therefore, both the incidence angle and the wavelength of light can be used as the measurement parameter [12].

The three fundamental techniques for exciting surface plasmon resonance are prism coupling, waveguide coupling, and grating coupling: the most widely used is Kretschmann's method, which makes use of a prism coupler and thin metal film [10].

SPR-based sensors measure changes in the binding-induced refractive index, and so have the advantage that they do not require analyte labelling. As SPR by itself is not a selective technique, molecular recognition components (usually a thin gold or silver nanometer layer of metal or particle cluster array) are often immobilised on the sensing surface in order to increase performance [12]. For example, in order to detect an interaction, the ligand is immobilised on the sensing surface while its binding partner (the analyte) is in solution: when the analyte and ligand bind, the accumulation of protein on the surface leads to an increase in the refractive index that can be measured in real time and is plotted in the form of response or resonance units (RUs; one RU represents the binding of approximately 1 pg protein/mm^2^) against time (a sensorgram) [13].

SPR sensing can be used to evaluate protein-ligand, protein-protein, or nucleotide hybridisation events. The most recent clinical applications of SPR biosensors include evaluating the osteogenic differentiation of mesenchymal stem cells on the basis of the differences in the optical properties of the cell surface caused by specific antigen-antibody binding [14]; quantitatively measuring the concentrations of fetal fibronectin (a biomarker predicting the risk of pre-term birth) in the cervicovaginal secretions of pregnant women [15]; and detecting serum human epididymis secretory protein 4 (a biomarker that plays an important role in the early diagnosis of ovarian cancer) in blood samples [16].

### 1.2. Fluorescence Resonance Energy Transfer

Förster or fluorescence resonance energy transfer (FRET) is an imaging technique in which a donor fluorophore transfers its energy to a neighbouring acceptor fluorophore, thus causing the acceptor to emit fluorescence at its characteristic wavelength [17]; the emitted light can be captured by a sensor and measured.

The successful transfer of energy between donor and acceptor requires the following three conditions: the donor and acceptor probes must be in close proximity (1–10 nanometres), have a favourable dipole-dipole alignment, and share significant spectral overlap (at least 30%) [18]. Because of this last condition, the number of florescent tag proteins that can be used in combination with each other is limited. Pairs of organic fluorescent tags are often defined as “FRET pairs”, and the most widely used include cyan fluorescent protein (CFP) and yellow fluorescent protein (YFP), FITC and rhodamine, CFP and dsRED, BFP and GFP, Alexa488 and Cy3, GFP (or YFP) and dsRED, Cy3 and Cy5, Alexa488 and Alexa555 [19].

The structure of a FRET biosensor usually includes an acceptor fluorophore, a donor fluorophore, a ligand domain, a sensor domain, and linkers connecting each domain, and it may be classified as intermolecular (or bimolecular) or intramolecular (or unimolecular), both of which have advantages and disadvantages [20]. Intermolecular FRET biosensors consist of two molecules, the first including the acceptor fluorophore and ligand (or sensor) domain, and the second donor fluorophore and sensor (or ligand) domain. They are particularly useful for detecting protein-protein interactions in cells, but are difficult to use routinely because the quantification of FRET requires careful correction of the bleed-through of donor fluorescence into the FRET channel, and the cross-excitation of acceptor fluorophores. Intramolecular FRET biosensors carry all of their components in a single molecule and, unlike intermolecular biosensors, are easy to use [17]. FRET is most frequently measured in one of four ways: spectral bleed-through correction, spectral imaging, acceptor photobleaching, and time-resolved fluorescence [18].

Since the emergence of the fluorescence protein in 1992, fluorescence-based biosensors have been widely used to monitor and identify living cell dynamics in real time. FRET donor and acceptor fluorophores can be conjugated to form multiple biomolecules, thus allowing the design of functional assays that can be used to study various types of binding, including protein-protein binding, antigen-antibody binding, ligand-receptor binding, DNA or RNA hybridisation, and DNA or RNA-protein binding [19].

For example, Waterhouse *et al*. have developed a FRET-based biosensor assay that is capable of detecting Bcr-Abl and epidermal growth factor receptor activity and its inhibition by tyrosine kinase inhibitors *in vivo*[21]. A FRET-based assay has also been developed to monitor the ligand-dependent interactions of an estrogen receptor isoform in breast cancer cells [22].

DNA- or RNA-based FRET probes are used in many *in vitro* and *in vivo* applications with the aim of monitoring various types of DNA and RNA reactions, such as polymerase chain reactions (PCRs), hybridisation, ligation, cleavage, recombination, and synthesis. FRET-based assays have also been used to monitor the status of DNA methylation [19].

### 1.3. Bioluminescence Resonance Energy Transfer

Bioluminescence resonance energy transfer (BRET) resembles FRET in many aspects but does not require an external light source for donor excitation. In this case, the donor is usually an enzyme that emits light during the catalysis of the oxidation of its substrate (such as the luciferase enzyme), and the acceptor is a fluorescent protein that absorbs the energy of the donor and emits light at a longer wavelength [23]. The change in the luminescence ratio can be quantitatively analysed. BRET was first used in 1999 to investigate the dimerisation of cyanobacterial circadian clock proteins in bacterial culture [24].

The fluorescent proteins used as acceptors are derivates of green fluorescent protein (GFP) from the jellyfish *Aequorea victoria*, which was first cloned by Prasher *et al*. [25]. The donor in the original BRET description was Renilla luciferase, and the receptor was GFP. Both can be genetically fused to proteins of interest for use in BRET or other applications. Since its initial description, BRET has evolved into a number of new forms using novel donor and acceptor pairs: the most frequently used BRET donors include DeepBlueC and firefly luciferase (Fluc) from *Hotaria parvula*, whereas many GFP mutants with varying spectral properties are available as acceptors [23].

“Nano-lantern”, a chimera of enhanced *Renilla* luciferase and Venus, is a recently developed fluorescent protein whose highly efficient BRET makes it the brightest luminescent protein so far available; it can used to enable the real-time imaging of intracellular structures in living cells with greater spatial resolution, and sensitively detects tumours in freely moving, unshaved mice [26].

Energy transfer occurs when the proteins of interest interact to bring the donor and acceptor into close proximity: RET efficiency is inversely proportional to the distance between the donor and acceptor molecules, varying with the sixth power of the distance [24]; this dependence on distance makes BRET a powerful means of identifying and imaging protein-protein interactions.

Like FRET, BRET is a broadly applicable method and has an ever-increasing number of applications. Moreover, as BRET does not require an external light source for donor excitation, it has additional advantages over FRET: it does not photodamage cells or photobleach the fluorophores; it has no auto-fluorescence background; and the acceptor is not directly excited [23].

Two examples of the most recent applications of BRET biosensors are the real-time monitoring of cytokine IL-1β processing in macrophages [27], and the analysis of agonist-induced changes in the compartmentalisation of type I angiotensin receptors, including their internalisation or lateral movement between plasma membrane compartments in response to stimulation [28].

## 2. Clinical Applications

Leland C. Clark Jr., who published his definitive paper on the oxygen electrode in 1956, can be considered the father of the biosensor concept [2]. Since then, much progress has been made, and biosensors are now used in many fields: in the food industry, they can detect the presence of harmful bacteria in alimentary products [29]; in forensics, they can help investigators identify human blood at a crime scene [30]; in counter-terrorism, they can detect explosives and explosive-related compounds [31]. However, this review will only consider their medical applications, which account for more than 80% of all commercial biosensor-based devices [32]; the following paragraphs describe some examples of their use in endocrinology, microbiology and oncology.

### 2.1. Endocrinology

The main clinical application of biosensors is to measure blood glucose levels in diabetic patients. Diabetes mellitus, an endocrine disorder affecting carbohydrate metabolism, is a major health problem in most developed societies, and its prevalence is steadily increasing due to sedentary lifestyles, changes in eating habits, and obesity. Various laboratory tests are used to diagnose and manage patients with diabetes, but the most important is measuring glycemia (blood glucose concentrations) [33].

Most glucose biosensors use enzymes known as oxidoreductases (glucose oxidase and glucose dehydrogenase), and they are usually electrochemical (amperometric). They are based on the oxidation of β-D-glucose by molecular oxygen into gluconic acid and the hydrogen peroxide catalysed by the immobilised glucose oxidase enzyme [34]. During the course of the reaction, the redox co-factor flavin adenine dinucleotide (FAD) works as the initial electron acceptor. It is first reduced to FADH_2_, and then regenerated by reacting with oxygen to form hydrogen peroxide. Hydrogen peroxide is oxidised, and the number of electron transfers during this oxidation (which is proportional to the number of glucose molecules in the sample) can be recognised by an electrode, or the glucose molecules can be quantified by measuring oxygen consumption [33].

The first biosensor for measuring glucose levels was constructed in 1962 by Clark and Lyons, who coupled glucose oxidase to an amperometric electrode in order to measure oxygen pressure: the electrode sensed the reduction in oxygen pressure caused by the enzyme-catalysed oxidation of glucose in the test solution, which was proportional to the reduced glucose concentration in the sample [35].

The first commercially successful glucose biosensor appeared in 1975; it was an analyser for the direct measurement of glucose based on the amperometric detection of hydrogen peroxide according to Clark's technology; however, its use was confined to clinical laboratories because of its expensive platinum electrode [33].

The first-generation glucose biosensors used a natural oxygen substrate and detected the production of hydrogen peroxide, but they were characterised by technical disadvantages such as the interference of endogenous electroactive species (such as ascorbic acid, uric acid, and certain drugs) or the restricted solubility of oxygen in biological fluids; moreover, the amperometric measurement of hydrogen peroxide required a high operative potential in order to increase selectivity. In the second-generation devices, oxygen was replaced by redox mediators (non-physiological electron acceptors such as ferrocene, thionine, methylene blue and methyl viologen), which had the capacity to transfer electrons from the enzyme to the surface of the working electrode [36]. Instead of hydrogen peroxide, this system forms a reduced mediator that is then re-oxidised at the electrode to provide an amperometric signal [37].

The third-generation glucose biosensors do not use reagents but are based on direct transfer between the enzyme and electrode, and have led to implantable, needle-type devices for the continuous *in vivo* monitoring of blood glucose. The electrode in this type of biosensor can perform direct electron transfers using organic conducting materials (such as tetrathiafulvalene-tetracyanoquinodimethane) based on charge-transfer complexes. This avoids the need to use highly toxic mediators, and improves the selectivity of the device; however, only a few enzymes show direct electron transfer on normal electrode surfaces [33].

Biosensors are currently being developed that use the recently discovered cellobiose dehydrogenase produced by the ascomycete *Corynascus thermophilus* (CtCDH) as an alternative to glucose-oxidising enzymes. CtCDH catalyses the oxidation of mono- and disaccharides such as glucose and maltose, and can transfer the electrons gained by the substrate oxidation directly to the electrode surface. Preliminary results show that this new kind of biosensor has a wide linear concentration range and a low limit of detection; furthermore, it is highly selective and stable, and is therefore likely to be successfully applied to monitoring glycemia in patient blood samples [38].

Glucose biosensors account for 90% of the global biosensor market [8] and, since their introduction, diabetic patients have been able to monitor their blood glucose levels easily at home, and consequently self-administer insulin doses as required.

### 2.2. Microbiology

Common bacterial urinary tract infections (UTIs) are not only a major cause of patient morbidity, but also lead to high healthcare costs, many of which are due to the processing of urine specimens by clinical microbiology laboratories; moreover, there is a typical delay of 2–3 days between culturing urine samples and obtaining the results of antimicrobial susceptibility testing [39]. For this reason, clinicians start antibiotic therapy empirically, which can lead to adverse drug reactions and the development of bacterial resistance. It would therefore be very useful to have a rapid molecular means of detecting and identifying urinary bacterial pathogens [40], which can be done by using electrochemical DNA biosensors in which the sensory receptor is a layer of oligonucleotide probes and the sensory input is detected by an electrochemical transducer [5].

Liao *et al*. used this class of biosensors to detect and identify pathogens in clinical urine specimens molecularly by means of an array of 16 electrochemical sensors, each consisting of three single-layer (working, auxiliary and reference) gold electrodes. They inserted a capture probe specific for a clinically relevant bacterial pathogen (e.g., *Proteus mirabilis*, *Escherichia coli*, *Enterocococcus* spp., *Pseudomonas aeruginosa*, and the *Klebsiella-Enterobacter* group) into each working electrode and, in practice by means of single-step bacterial lysis, obtained the bacterial 16S rRNA target. This was then hybridised to the biotin-modified capture probe on the sensor surface and a fluorescein-modified detector probe (the capture probe anchored the target to the sensor, and the detector probe recognised the target bound on the sensor surface). A horseradish peroxidise (HRP)-conjugated anti-fluorescein antibody was linked to the detector probe in order to detect target-probe hybrids. The reaction catalysed by HRP (the reporter enzyme) was amperometrically measured at a fixed potential between the working and reference electrodes and the current amplitude indirectly reflected the number of target probe-reporter enzyme complexes anchored to the sensor surface.

Using this system, more than 2,500 species-specific uropathogenic bacteria can be detected in only 45 minutes instead of the two days necessary for microbial cultures. The sensor array was highly sensitive in directly detecting gram-negative bacteria in blinded clinical urine specimens: it identified 98% of the Gram-negative bacteria for which species-specific probes were available without the need for the nucleic acid amplification or purification required by other classical techniques such as polymerase chain reaction (PCR) [40].

Mohan *et al*. developed a biosensor array capable of simultaneously detecting two different targets –pathogen 16S rRNA and host lactoferrin (LTF, a surrogate marker of pyuria)—in urine samples from patients with spinal cord injuries, who are at high risk of developing complicated urinary tract infections due to structural and physiological impairment of bladder emptying, vesicoureteral reflux, and the need for in-dwelling catheters. For this purpose, a set of oligonucleotide probes targeting common (*E. coli*, *P. mirabilis*, *P. aeruginosa* and *Enterococcus spp.*) and less common uropathogens (*Serratia*, *Providencia*, *Morganella* and *Staphylococcus spp*) was developed and optimised for hybridisation at 37 °C in order to facilitate integration with protein biomarker target binding, which usually occurs at this temperature.

A single 16-sensor array was used for both determinations: the surface of 11 of the sensors in the array was functionalised with capture probes for pathogen identification and the remaining five were functionalised with capture antibodies for LTF detection. Sandwich hybridisation of the capture and detector oligonucleotides to bacterial 16S rRNA was used to detect the pathogen, whereas an electrochemical sandwich assay based on capture and detector antibodies to lactoferrin was used to detect the pyuria marker. Both sandwich assays were coupled to a horseradish peroxidase-based redox reaction, giving rise to a measurable electrical signal. The analytical performance of this integrated assay was found to be promising: pathogen detection was highly specific (97%) and sensitive (89%), but LTF levels did not predict the need for antibiotic therapy in the subset of patients tested, and so further development may include the identification of additional biomarkers specific for infection in catheterised patients [39].

### 2.3. Oncology

Over the last fifty years, the incidence of cancer has increased and oncological diseases have become some of the most lethal diseases for humans [41]. In 2012, there were an estimated 3.45 million new cases and 1.75 million deaths due to cancer in Europe alone. The most common diseases are female breast cancer, followed by colorectal, prostate and lung cancer [42].

As an early diagnosis is even more crucial for the survival and positive prognosis of oncological patients than patients with other conditions, sensitive and specific means of diagnosing cancer early are strategically necessary.

One of the most widely used methods consists of searching for specific biomarkers, defined by the National Cancer Institute as “biological molecules found in blood, other body fluids, or tissues that are a sign of a normal or abnormal process or of a condition or disease; a biomarker may be used to see how well the body responds to a treatment for a disease or condition” [3,7].

Biomarkers are involved in many steps of cancer management: they can be used not only for diagnosis, but also for assessing risk and prognosis, monitoring therapeutic efficacy, and sometimes as therapeutic targets when they directly contribute to tumour growth [43]. In Table 1, a summary of major cancer-specific biomarkers is provided.

Biomarkers can be detected using biosensors that are usually based on the highly specific molecular recognition of antibodies and antigens whose interactions give rise to immunocomplexes [44], and have the advantages that they are cheaper, faster and more flexible than other methods, and also allow the use of multi-target analyses and automation [45].

#### 2.3.1. SPRI Biosensor for Proteasome

In mammalian cells, the enzymatic complex known as 20S proteasome catalyses the intracellular degradation of mutated or damaged proteins, viral proteins, and many of the short-lived proteins involved in cell cycle progression, apoptosis, and signal transduction [46].

**Figure 2.**
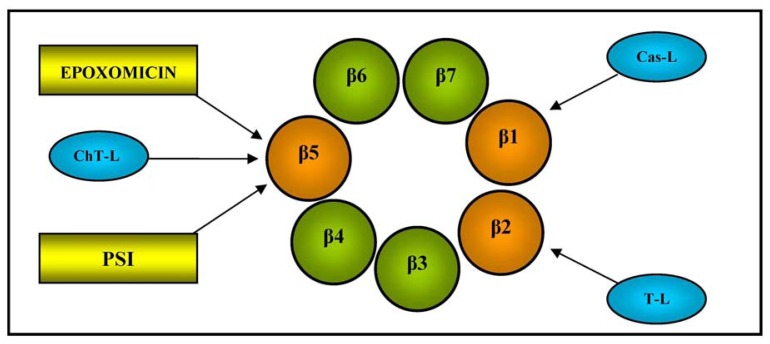
Structure and function of the β-ring of 20S proteasome. Schematic illustration of a 20S proteasome β-ring showing the subunits responsible for caspase-like (Cas-L), trypsin-like (T-L) and chymotrypsin-like (ChT-L) activities, and the site of action of inhibitors (the synthetic peptide PSI and epoxomicin).

It has a mass weight of 700 kDa and a cylindrical structure consisting of two outer α-rings and two inner β-rings, each with seven different subunits (α1-7 and β1-7). The function of the outer α subunits is to control the entry of substrate proteins into the central catalytic zone and bind the regulators, whereas the three inner β1, β2, and β5 subunits contain a catalytically active N-terminal threonine (Thr) residue, and are respectively responsible for caspase-like (Cas-L), trypsin-like (T-L) and chymotrypsin-like (ChT-L) activities (see Figure 2 for a schematic illustration of 20S proteasome β-ring); the last is the rate-limiting step in the degradation of intracellular proteins [47].

Plasma proteasome antigen levels or enzymatic activities are biomarkers used for the prognosis and monitoring of patients with various cancers or other diseases, and bortezomib is a proteasome inhibitor that is currently widely used in clinical practice, mainly in the treatment of multiple myeloma [48].

Gorodkiewicz *et al*. developed a method of determining 20S proteasome levels using a surface plasmon resonance imaging (SPRI) biosensor and the highly selective interaction between the proteasome's catalytic β5 subunit and immobilised inhibitors (the synthetic peptide PSI and epoxomicin) [49]. SPRI is a label-free technique that combines the previously described principle of SPR with an imaging system that allows the simultaneous monitoring of up to hundreds of molecular interactions; during the analysis, the reflected light is monitored by a detector system (usually a charge-coupled device) at a fixed angle defined just above the resonance angle [11].

The study used an array of 9 × 12 gold chips allowing 12 simultaneous SPRI measurements of nine different solutions, and the inhibitors (PSI or epoxomicin) were immobilised on the chip surfaces onto which drops of plasma from nine healthy adults and nine patients with acute leukemia were transferred for 10 min. The SPRI signals were obtained twice: after the immobilisation of the inhibitor, and after its interaction with the 20S proteasome contained in the samples. The difference between the signals obtained before and after the interaction gave the exact SPRI signal, which was proportional to the coupled biomolecules and indirectly proportional to the proteasome concentration in the analysed plasma. The biosensor was found to be suitable for quantitative determinations, and the results showed that all of the patients had increased plasma 20S proteasome concentrations, which were up to 15 times higher than those found in the plasma of the healthy subjects [49].

#### 2.3.2. Biosensors for the Simultaneous Detection of Different Tumour Markers

Some biosensors have been constructed in order to allow the simultaneous detection of different tumour markers. One was developed by Wilson and Nie, and allows the simultaneous measurement of α-fetoprotein, ferritin, β-human chorionic gonadotropin, carcinoembryonic antigen, cancer antigen 125, cancer antigen 15-3 and cancer antigen 19-9 levels. It consists of an array of immunosensing electrodes, each of which contains a different immobilised antigen and is capable of measuring one of the seven specific tumour markers by means of a competitive, electrochemical, enzyme-based enzyme-linked immunosorbent assay (ELISA) [50].

A second was designed by Wu *et al*., and detects α-fetoprotein, β-human chorionic gonadotropin, carcinoembryonic antigen and cancer antigen 125 on the basis of the competitive immunoreaction between the four tumour markers immobilised in a redox mediator-grafted carbon electrode array and the corresponding horseradish peroxidase-labelled antibodies. The immunosensor array contained eight graphite working electrodes, one graphite auxiliary electrode and one reference electrode, and was connected to a flow injection system; phosphate buffered saline with hydrogen peroxide was used as carrier solution. The antigens were immobilised on the working electrodes using chitosan grafted with molecules of the redox mediator toluidine blue O (TBO) as the immobilisation matrix. The presence of the redox mediator increased the sensitivity of the immunoassay by accelerating the electrochemical reduction of the hydrogen peroxide catalysed by horseradish peroxidase (HRP)-labelled monoclonal antibodies. The mediator-catalysed enzymatic response to hydrogen peroxide was measured amperometrically, and decreased proportionally with the analyte concentrations in the samples. This multi-analyte immunosensor array was found to be capable of testing 60 samples in an hour, with a sensitivity that is sufficient for clinical purposes [51].

#### 2.3.3. Biosensor for Detecting a Protein Marker of Myelodysplastic Syndromes

Myelodysplastic syndromes (MDS) are clonal disorders of hematopoietic stem cells that are clinically characterised by ineffective hematopoiesis, which leads to peripheral blood cytopenias and the possible transformation into acute myeloid leukemia (AML) [52]. MDS are currently diagnosed using invasive procedures such as bone marrow aspiration, core biopsies and peripheral blood counts [53], but it would be useful to be able to use the high-throughput screening of molecular markers in order to improve the diagnostic and prognostic characterisation of such a heterogeneous disease.

A number of protein markers of MDS have recently been identified, and it has been reported that the soluble form of vascular endothelial growth factor receptor 1 (sVEGFR-1) is over-expressed in patients with MDS and AML [54]. VEGFR-1 is a transmembrane protein with tyrosine kinase activity, and may be soluble or membrane-bound [55]. The signalling pathway mediated by the VEGFR-1 receptor is triggered by the binding of its high-affinity ligand, the cytokine vascular endothelial growth factor A (VEGF-A), which initiates important processes such as cell proliferation, endothelial mitogenesis, angiogenesis and vasculogenesis [56].

The current methods of detecting sVEGFR-1 are immunological (ELISA) and the use antibodies to capture the protein marker of interest with fluorescent labels (or enzymatic amplification) revealing antibody-protein binding. They are effective but their disadvantages include the need for high-affinity antibodies, which are difficult and expensive to produce, and their limited ability to detect complexes of sVEGFR-1 and other molecules [57].

Pimková *et al*. developed a label-free method for detecting plasma sVEGFR-1 through its physiological ligand VEGF-A by means of a surface plasmon resonance (SPR) biosensor. VEGF-A is covalently immobilised on the surface of the sensor, and the plasma is transferred to the sensing area using dispersionless microfluidics, a sequential injection of the sample and a special buffer. Microfluidics allows rapid switching between the plasma and buffer (thus eliminating dispersion and intermixing before the sample reaches the sensing area), and the sequential injection makes it possible to eliminate the non-specifically adsorbed entities from the surface without affecting the analyte molecules bound to the immobilised ligand. These techniques minimise the non-specific sensor response and allow the detection of low concentrations of analytes, even in complex samples.

The binding of the analyte (plasma sVEGFR-1) to the biomolecular recognition element (VEGF-A) immobilised on the surface of the sensor causes a change in the local refractive index, and the amount of captured analyte is quantified by measuring the change in the resonant wavelength.

It has been shown that this system has a detection limit of 25 ng/mL, and so this study may be a model for the future development of protein microarrays for the diagnosis of MDS based on protein-protein interactions under physiological conditions [52].

## 3. Applications in Oncohematology

This section describes the biosensors that have been developed as a means of optimising the diagnosis and treatment of patients with hematological malignancies (such as leukemias, lymphomas and multiple myeloma) that are highly curable but clinically extremely demanding and characterised by very high management costs [58]; for these reasons, the development of techniques aimed at improving diagnosis, prognosis and therapeutic monitoring is an urgent need.

### 3.1. Biosensor for Diagnosing Leukemia

An early and precise diagnosis is essential for the effective and successful treatment of all diseases, and cancer is no exception. As current diagnostic methods are often expensive (because they require advanced instrumentation) and time consuming, more rapid and cost-effective methods would be very useful. To this end, Medley *et al*. developed a colorimetric assay based on aptamer-conjugated gold nanoparticles (ACGNPs) that is capable of distinguishing cancerous and non-cancerous samples [59].

Aptamers are oligonucleotide strands that bind to selected molecules with a high degree of selectivity and affinity; their diagnostic capacity is similar to that of antibodies [60]. For this assay, the aptamers were selected using the cell-SELEX technique, in which live whole cells rather than single molecules are the target [61]. Colorimetric detection technology uses the optical properties of gold nanoparticles and plasmon resonance, which measures the variation in absorption spectra and scattering profiles when two nanoparticles are near to each other, and leads to changes in the colour and absorption spectra of the sample [62]. ACGNPs can assemble on the surface of a specific type of cancer cell because of the target recognition of aptamers on the cell membrane, and this assembly causes surface plasmon resonance because the close proximity of the particles leads to a shift in their absorption spectra.

**Figure 3.**
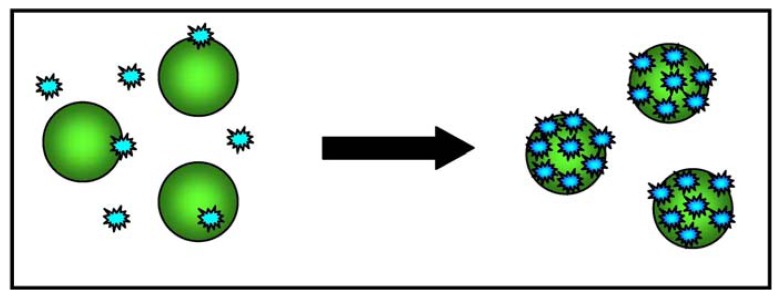
Schematic illustration of the ACGNP-based colorimetric assay. When the gold nanoparticles are near each other, their absorption spectra and scattering profiles vary, leading to a change in the colour and absorption spectra of the sample that is visible to the naked eye.

A subsequent study evaluated whether the ACGNP approach can differentiate target cells (CCRF- acute leukemia cells from the CEM cell line) and control cells (Ramos, derived from a patient with Burkitt's lymphoma). To this end, an aptamer sequence with a high degree of affinity and selectivity for CCRF-CEM acute leukemia cells was conjugated to gold nanoparticles, and the absorption spectra of five samples containing different amounts of target and control cells were determined. It was found that the assembly of the ACGNPs around the target cells caused an increase in the absorption and scattering of the solution that was proportional to the number of target cells in the sample. The sample with the largest number of target cells took on a darker colour, whereas the sample with control cells remained colourless, thus allowing the detection of target cells with the naked eye (when there were at least 1000 target cells in the sample). Figure 3 provides a schematic illustration of the ACGNP-based colorimetric assay.

For the purpose of more sensitive detection, the samples were also analysed using a microplate reader, and the results showed that the absorption spectra correlated well with the colorimetric results as the samples with more target cells absorbed light more intensely.

The same excellent results in terms of sensitivity and selectivity were obtained when aptamers selective for the Ramos cell line (target cells) were used with CCRF-CEM acute leukemia cells as the negative control, thus demonstrating the system's ability to identify different cell targets.

The cell-SELEX method has also been used to generate specific aptamers for small-cell lung and liver cancer, thus suggesting that it is feasible to develop colorimetric assays for detecting these diseases. The next step is to test the performance of this colorimetric assay using complex samples (of which blood will be the paradigm) and, if the results are satisfactory, the assay will become a very welcome additional means of detecting cancer cells because of its rapidity, cost-effectiveness and sensitivity [59].

### 3.2. Biosensor for Acute Leukemia Immunophenotyping

The CD33 antigen is a transmembrane protein present on the surface of myeloid, megakaryocytic, erythroid and multipotent progenitor cells that is used as a cell surface marker for the clinical diagnosis and therapeutic targeting of acute myeloid leukemia. Cell surface markers are usually detected by means of flow cytometry, using antibodies specific for the antigen of interest [63]. However, this method is time consuming and has other disadvantages, such as the need for fluorescence labelling, high instrumentation costs, and a low analytical throughput [64], some of which may be overcome by using biosensors.

Fang *et al*. developed a novel multichannel biosensor system combining the principle of SPR biosensing with spectral imaging technology, and compared its efficacy in detecting AML cells expressing antigen CD33 with that of flow cytometry. In this experiment, eleven whole marrow aspirate specimens from patients with acute myeloid leukemia were diluted in a sterile phosphate buffer solution, and then dropped by means of a pipette onto the different sensing spots of a sensor chip array on which anti-CD33 monoclonal antibodies (mAbs) had been previously immobilised; two spots were left without a specimen for reference purposes. After 30 minutes of binding interaction with the anti-CD33 mAbs, the specimen solutions were removed and it was found that the resonance wavelengths had increased as a result of the change in the refractive index caused by the affinity binding of the leukemia cells to the mAbs. In practice, the difference in wavelength was used as a quantitative measure of the changes in the refractive index of the sensor surface.

The results obtained using this system were similar to those obtained by means of flow cytometry, which suggests that the system could be used to detect CD33+ cells in marrow aspirates taken from patients with acute myeloid leukemia. Moreover, in comparison with flow cytometry, the system was cheaper and easier to construct because it did not require cell separation and labelling, and may therefore become a convenient alternative when immunophenotyping acute leukemias [65]. However, the approach has two major drawbacks: the complete loss of morphological parameters, and the theoretical difficulty of developing biosensors that can detect multiple cell surface markers (neither of which hamper the use of flow cytometry).

### 3.3. SPR Biosensor Assay for Asparaginase Antibodies

l-Asparaginase is an enzyme that catalyses the hydrolysis of l-asparagine (an endogenous amino acid necessary for the normal course of the cell cycle in cells such as lymphoblasts) into aspartic acid and ammonia. Unlike most human cells, lymphoblasts cannot compensate for the l-asparagine deficiency induced by l-asparaginase by means of an alternative pathway and, as this leads to blastic cell apoptosis, l-asparaginase has been successfully used in multidrug therapeutic schedules for children and adults with acute lymphoblastic leukemia (ALL) [66].

Like other protein therapies, treatment with bacterial asparaginase proteins can lead to immune responses and the development of human anti-bacterial antibodies. The presence of antibodies in patients with hypersensitivity reactions to asparaginase preparations has been associated with a more rapid clearance of enzymatic activity and, consequently, a reduction in the half-life of native or pegylated *Escherichia coli* formulations, thus leading to poorer outcomes in a subgroup of ALL patients [67]. Once patients have presented an immune response, they can be treated with a non-cross-reacting asparaginase, and so monitoring the effects of asparaginases and the presence of antibodies may help therapeutic decision making.

Avramis *et al*. developed an SPR biosensor assay to determine the presence of antibodies in serum taken from ALL patients who developed a clinical allergy to native or pegylated asparaginase (pegaspargase) treatment (n = 84) and control volunteers (n = 6), and compared the results with those obtained using an ELISA. The biosensor assay was protein based and had human *E. coli* asparaginase, pegaspargase and Erwinase proteins covalently coupled to the carboxy-methylated dextran matrix of a sensor chip. The patients' serum was diluted with running buffer and then injected over all four sensor surfaces for one minute at a rate of 30 mL/minute.

This assay detected antibody binding to immobilised asparaginase protein, thus revealing the presence of neutralising IgG. The results showed that it accurately detected the antibody more specifically and rapidly than the ELISA (300 s *vs.* 24 h). Furthermore, unlike the ELISA, it determined the antibody subtype (IgG) and whether the antibody was neutralising or not [68].

### 3.4. Bioluminescent Microbial Biosensors for Assessing the Ara-C Sensitivity of Leukemia Cells

Ara-C (cytosine arabinoside) is a cytotoxic nucleoside analogue that is widely used to treat acute leukemias; however, some patients do not respond or become drug resistant [69], whereas others seem to be very sensitive and may achieve long-term remission at lower drug doses. The absence of a pre-screening test to evaluate the potential response to Ara-C exposes non-responders to possibly inefficacious treatment, and hypersensitive patients to over-treatment [70].

From a pharmacokinetic point of view, Ara-C is a pro-drug as it has to be activated in cells by means of phosphorylation catalysed by the deoxycytidine kinase (dCK) enzyme, whereas it is mainly deactivated by the enzyme cytidine deaminase (cdd) [71]. The active form of Ara-C triphosphate (Ara-CTP) acts by interfering with DNA synthesis: once incorporated into DNA strands, it leads to chain termination and the arrest of DNA synthesis [72].

Alloush *et al*. created a pyrimidine-requiring *cdd*-deficient *Escherichia coli* mutant that expressed the human *dCK* gene and was made luminescent by the introduction of the *luxCDABE* operon, which was used to determine Ara-C uptake and phosphorylation by leukemic cells [73].

Any alteration in the cell metabolism of bioluminescent bacteria causes a change in light emission, which is increased in the case of DNA damage [74].

**Figure 4.**
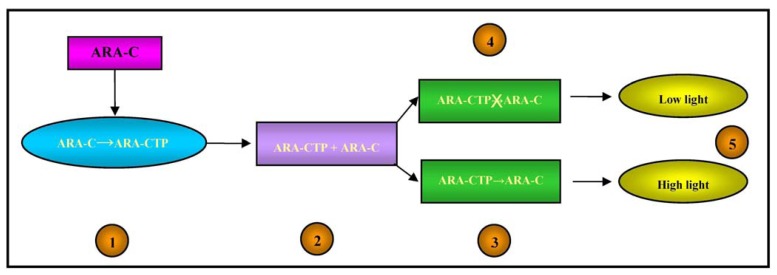
Schematic illustration of the test. **1**: Mononuclear cells from AML patients were incubated with Ara-C at a final concentration of 25 micromol/L (equivalent to the standard *in vivo* dose of 200 mg/m^2^/day in a subject weighing 80 kg), washed and lysed; **2**: The subsequent cell lysate (containing both Ara-C and Ara-CTP) was exposed to the bacterial biosensor in the presence or absence of alkaline phosphatase (Ara-CTP does not enter the reporter bacteria and shows no increase in light output unless alkaline phosphatase is added at the start of the assay); **3**: In the presence of alkaline phosphatase, Ara-CTP is converted to Ara-C, which enters the bacteria and allows the generation of bioluminescence; **4**: In the absence of alkaline phosphatase, Ara-CTP cannot be converted into Ara-C and therefore cannot enter the bacteria; **5**: The ratio between the light in the presence/absence of alkaline phosphatase is directly proportional to the concentration of Ara-CTP in the patient's blasts, which is representative of the conversion of Ara-C into Ara-CTP.

The intracellular concentrations of Ara-CTP and its pharmacological effects on DNA were therefore detected by means of the light output from the bacterial biosensor, as illustrated in Figure 4.

Using acute myeloid leukemia cell lines with a known response to Ara-C, the biosensor results closely correlated with those obtained using colony forming units/blast clonogenic assays; furthermore, they were obtained in eight hours rather than three days, thus making the system more suitable for routine screening. If fully validated, the biosensor-based assay could be used routinely in the clinical setting to predict the sensitivity of leukemic cells to Ara-C before exposing patients to chemotherapy, and promote the development of tailored treatment strategies [73].

A second analogous bioluminescent bacterial biosensor for the detection of intracellular levels of Ara-CTP has been recently described by Andersen *et al*. [75]; it predicted the response of seven leukemic cell lines with different known sensitivity to cytarabine when the drug was used in monotherapy or combined with fludarabine. The results were confirmed by means of Ara-CTP quantification using high-performance liquid chromatography.

### 3.5. FRET Biosensor for Assessing the Efficacy of Imatinib in Patients with Chronic Myeloid Leukemia

Chronic myeloid leukemia (CML) is a myeloproliferative disorder that accounts for a large fraction of adult leukemias and is cytogenetically characterised by the presence of the Philadelphia chromosome (Ph), which originates from a reciprocal translocation between the long arms of chromosomes 9 and 22 and leads to the formation of a novel fusion gene called bcr-abl, the product of which is the oncogenic chimeric kinase BCR-ABL that is involved in the pathogenesis of CML [76].

Tyrosine kinase inhibitors (TKIs, of which the first was imatinib mesylate) act by blocking the ATP binding site of BCR-ABL, and are key drugs for the treatment of CML and other malignancies such as gastrointestinal stromal tumours whose growth depends on the activity of specific kinases [77]. However, there is growing concern about the emergence of resistance to imatinib and other TKIs attributable to amplifications or mutations in the bcr-abl gene, clonal evolution, and the heterogeneous activity of BCR-ABL on CML cells. The main substrate of BCR-ABL in human cells is CrKL, a Crk adaptor molecule that mediates a multitude of pathophysiological signalling pathways that regulate various cell functions [78]. It includes one src homology 2 (SH2) domain, two SH3 domains, and a tyrosine residue that is phosphorylated by cellular tyrosine kinases. In human CML cells, CrkL is constitutively phosphorylated and plays an important role in oncogenic signal transduction [79]. For this reason, the level of CrkL phosphorylation revealed by immunoblotting has been used as a marker of BCR-ABL activity and drug response [80].

**Figure 5.**
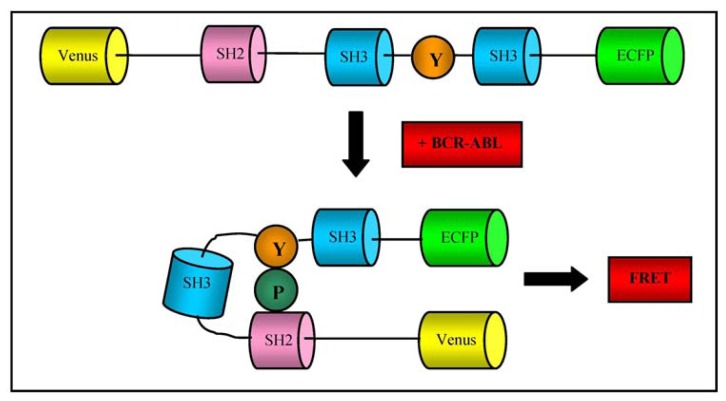
Schematic representation of phosphorylated and non-phosphorylated Pickles. P and Y respectively represent a phosphate group and a tyrosine residue. Upon Y phosphoryl-ation, the SH2 domain binds to the phosphorylated tyrosine, which increases the efficiency of FRET from ECFP to Venus.

Mizutani *et al*. developed an original fluorescence resonance energy transfer (FRET) system for determining BCR-ABL activity and its inhibition in response to imatinib treatment in CML cells obtained by aspirating the bone marrow of CML patients. They designed a protein called “Pickles” (phosphorylation indicator of CrkL en substrate) in which CrkL is sandwiched between Venus, a variant of yellow fluorescent protein, and enhanced cyan fluorescent protein (ECFP), so that intramolecular CrkL binding of the SH2 domain to phosphorylated tyrosine (Y207) increases FRET efficiency (as represented in Figure 5); when transfected into CML cells obtained by bone marrow aspiration, this biosensor was more sensitive in measuring BCR-ABL activity and its suppression by imatinib than other established methods, including western blotting and flow cytometry [81].

The authors also verified that nilotinib and dasatinib (two second-generation TKIs) were more potent than imatinib, and demonstrated that the FRET biosensor could detect a small amount of drug-resistant cancer cells mixed in a large cell population when associated with flow cytometry.

As these cells may be the main cause of CML relapse and therapeutic failure, Pickles could be used to quantify the presence of cells resistant to the different TKIs in bone marrow samples from CML patients, thus possibly helping clinicians to optimise TKI therapy and maximise the chances of success [82].

## 4. Future Perspectives

Recent advances in micro- and nano-scale technologies should soon make it possible to develop new biosensing applications [5]. The use of genomic and proteomic molecular tools that allow the profiling of different cancer cells will lead to new opportunities for the use of biosensors in tumour diagnosis and prognosis, and therapeutic monitoring and optimisation [45].

FRET biosensors will allow subcellular molecular signalling events to be visualised in real time, and improve the possibility of identifying novel targeting molecules and pathways [82].

BRET biosensors have been successfully used to study GPCR signal transduction: for example, they have clarified the real-time interactions between receptors and ligands, and variations in the amounts of second messengers. In the near future, it will be possible to understand GPCR signalling in more detail by developing new generations of BRET sensors [83].

The list of new sensors for analysing various signalling pathways and protein-protein interactions is rapidly growing. In the field of oncohematology, biosensors could be useful for identifying target pathways specific to each patient, and thus aid therapeutic decision making. A number of cancers, including blood malignancies, have been associated with the constitutive activation of proteins belonging to specific pathways, and inhibitors of these factors are progressively making their way from research laboratories to the bedside [84].

Examples of the numerous pathways potentially involved in the pathogenesis of hematological malignancies and possible key targets of already existing or new drugs include the pathways of VEGF receptor tyrosine kinase [85], the Janus kinase (JAK)/signal transducer and activator of transcription (STAT) [84], Wnt [86], RAS signalling [87], B-cell receptor (BCR) signalling [88], B-cell activating factor-receptor (BAFF-R) signalling [89], nuclear factor κB (NF-κB) [90], Notch signalling [91], GAB2 signalling [92], phosphatidylinositiol 3-kinase, AKT, mammalian target of rapamycin (PI3K/AKT/mTOR) signalling [93], p53 [94], histone deacetylase (HDAC) [95], and Fms-like tyrosine kinase 3 (FLT3) [96]. The use of biosensors to study the pathogenetic involvement of these pathways would support clinicians in choosing the best drug for individual patients and make an important step forward towards “personalised medicine”.

## Figures and Tables

**Table 1.** A list of cancer biomarkers.

**Cancer**	**Biomarkers**
Bladder	BAT, FDP, NMP22, HA-Hase, BLCA-4, CYFRA 21-1
Breast	CA15-3, CA125, CA27.29, CEABRCA1, BRCA2, MUC-1, CEA, NY-BR-1, ING-1
Colon and pancreas	CEA, CA19-9, CA24-2, p53
Esophagus	SCC
Gastric	CA72-4, CEA, CA19-9
Liver	AFP, CEA
Lung	NY-ESO-1, CEA, CA19-9, SCC, CYFRA21-1, NSE
Ovarian	CA125, AFP, hCG, p53, CEA
Prostate	PSA, PAP
Testicular	α-fetoprotein (AFP), β-human chorionic gonadatropin, CAGE-1, ESO-1
